# A randomised controlled trial investigating the ability for supervised exercise to reduce treatment-related decline in adolescent and young adult cancer patients

**DOI:** 10.1007/s00520-022-07217-w

**Published:** 2022-07-06

**Authors:** Claire Munsie, Jay Ebert, David Joske, Timothy Ackland

**Affiliations:** 1 School of Human Sciences (Exercise and Sport Science), The University of Western Australia, Perth, WA Australia; 2 WA Youth Cancer Service, Locked Bag 2012, Nedlands, WA 6009 Australia; 3 Sir Charles Gairdner Hospital, Perth, WA Australia; 4 School of Medicine, The University of Western Australia, Perth, WA Australia

**Keywords:** Exercise, Rehabilitation, Physical activity, Cancer, AYA, Physical function

## Abstract

**Introduction:**

Exercise is recognised as integral in mitigating a myriad negative consequences of cancer treatment. However, its benefit within adolescent and young adult (AYA) cancer cohorts remains relatively under researched, and caution should be taken in extrapolating outcomes from adult and paediatric populations given AYA distinctly different physiological and psychosocial contexts. This study sought to evaluate the impact of an exercise intervention on mitigating the expected decline in fitness, strength, physical functioning, and quality of life (QOL) in AYA undergoing cancer treatment.

**Methods:**

This prospective, randomised controlled trial (FiGHTINGF!T) allocated 43 participants (63% male, mean age 21.1 years) to a 10-week, multimodal, bi-weekly exercise intervention (EG) or control group (CG) undergoing usual care. Pre- and post-intervention assessments included cardiopulmonary exercise tests, one-repetition maximum (1RM) strength, functional tests, and QOL patient-reported outcome measures. Data were analysed via linear mixed models and regression.

**Results:**

While no significant group differences (*p* > 0.05) were observed, neither group significantly declined (*p* > 0.05) in any outcome measure over the 10-week period. No significant (*p*˃0.05) strength or functional improvements were observed in the CG, though the EG demonstrated significant improvements in their 1RM leg press (*p* = 0.004) and chest press (*p* = 0.032), maximal push ups (*p* = 0.032), and global QOL (*p* = 0.011). The EG reported a significant increase in fatigue (*p* = 0.014), while the CG reported significant positive changes in anxiety measures (*p* = 0.005).

**Conclusion:**

The exercise intervention produced superior improvements in strength and global QOL, compared with the CG. Regardless of group allocation, enrolment in the exercise study appeared to mitigate the treatment-related decline expected in AYA undergoing cancer treatment.

**Supplementary Information:**

The online version contains supplementary material available at 10.1007/s00520-022-07217-w.

## Introduction

Adolescents and young adults (AYA) present with physical, psychosocial, and practical needs that differ to their younger and older counterparts. In Australia, by definition, AYA are aged 15 to 25 years and account for approximately 1000 new cancer diagnoses annually [1, 2]. With advancements in treatments and supportive care, 5-year AYA survival rates for all cancers combined exceed 80% [2]. AYA cancer survivors live decades into survivorship burdened by the physical and psychosocial impacts of treatment [3, 4]. This may include lower rates of further education and employment, higher rates of depression and anxiety, lifelong fatigue, and poorer physical health, fitness, and quality of life (QOL) [5–7]. Therefore, it is increasingly important to understand the role of supportive therapies in mitigating the effects of cancer treatment in this cohort.

Previous research specific to paediatric and adult populations has demonstrated that exercise during cancer treatment can improve physical functioning, positively impact psychosocial well-being, and improve QOL [8–10]. However, more than 70% of AYA patients do not meet recommended physical activity guidelines for cancer patients while undergoing active treatment [11], with physical activity levels not recovering to pre-treatment levels in survivorship [12]. This lack of physical activity during and following cancer treatment often means that AYA have gross strength and fitness deficits compared with age-matched healthy peers.

High-quality randomised controlled trials (RCTs) involving exercise during cancer therapy in AYA are lacking [13]. Limited research has demonstrated cardiorespiratory fitness (CRF) [14] and walk distance [15] benefits in AYA as a result of exercise following treatment. These studies report clear evidence of patient deconditioning including reduced CRF and strength, suggesting a need for earlier intervention to prevent such decline [14, 15]. While not specific to AYA, research has reported a 5–26% decline in CRF during exposure to various chemotherapeutic regimens that may not recover following treatment [16, 17]. This reduction in CRF has been associated with higher symptom burden, as well as treatment-related toxicities [18]. Therefore, early intervention strategies are essential to reduce the burden of treatment side effects and establish healthy behaviours into survivorship.

With exercise now recognised as a necessary component of cancer treatment [19], its effect in the AYA setting must be rigorously investigated. Given the distinctly different physiological and psychosocial context of AYA comparative to younger and older cohorts, it is unwise to generalise findings from interventions in these cohorts to AYA. Therefore, the primary aim of the study was to investigate the impact of a 10-week supervised exercise intervention on the physical fitness (V0_2_ peak) of AYA undergoing cancer treatment. It further sought to assess the effects of the intervention on muscular strength, body composition, functional capacity, and psychosocial variables. It was hypothesised that the exercise intervention would be superior in limiting the rate of decline in physical fitness and functioning, compared with participants following a usual care regimen.

## Methods

### Participants

**Fig. 1:**
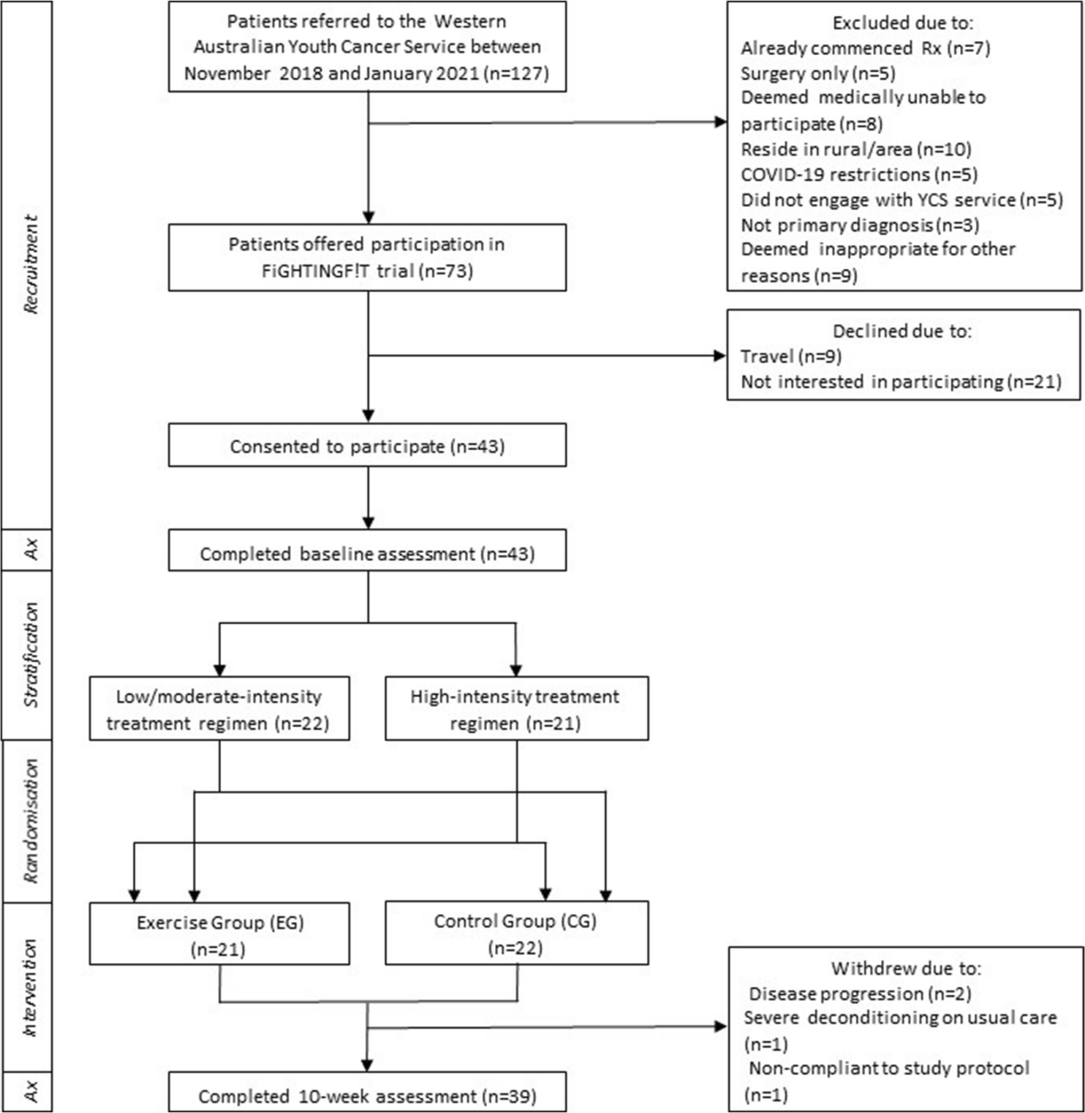
Flowchart of participant recruitment

A total of 127 AYA aged 15–25 years diagnosed with cancer were referred to the Western Australian Youth Cancer Service (WAYCS) from November 2018 to January 2021 and screened for eligibility to participate in the study (Fig. 1). Participants were eligible if their diagnosis was a primary malignancy, they were medically stable as per their treating clinician, and were assessed (within 2 weeks) prior to commencing systemic therapy (e.g. chemotherapy or combined chemotherapy and radiation). Participants were excluded if they were to undergo surgery only, had insufficient English competency or a cognitive impairment that would prevent them from participating, were medically unable to participate (as determined by their treating clinician), were pregnant or lactating, or had a life expectancy < 6 months. The study was approved by the University and Hospital Human Research Ethics Committees (HREC; RGS 714) and all participants and their treating clinician provided written informed consent. This trial was registered with the Australia New Zealand Clinical Trial Registry (ACTRN12620000663954).

### Experimental design

A prospective, single-centre RCT design was implemented (Fig. 1). Participants were stratified according to intensity of their cancer treatment regimen in order to reduce group bias: high-intensity versus low/moderate-intensity treatment, dictated by the likelihood of low blood counts and degree of myelosuppression anticipated from the treatment regimen (Supplementary Table 1). Randomisation was undertaken via a random assignment computer generator (sealed envelope) into one of the two treatment arms: exercise group (EG) or control group (CG). All participants underwent a series of assessments at baseline and 10 weeks and were contacted weekly over the 10-week period to monitor treatment-related toxicities and ensure adherence to the study protocol.

### Exercise and control interventions

The CG received general physical activity advice from the WAYCS Accredited Exercise Physiologist (AEP), the broader WAYCS team, and their treating clinical team as part of standard care; however, no exercise intervention was offered during the 10-week period. The EG involved twice-weekly exercise sessions for 10 weeks at a purpose-built gymnasium in a central hospital location. Sessions were supervised by an AEP and lasted approximately 60 min, individualised to the specific needs of each participant based on the results of their baseline assessments. Each session was of moderate intensity including a combination of aerobic, strength, and flexibility exercises as per the American College of Sports Medicine (ACSM) guidelines for cancer cohorts [10]. Sessions included a standardised aerobic warm up, a series of strength training exercises, and a low intensity aerobic cool down. Aerobic exercise was prescribed at 60–85% of maximal heart rate (220-age) [20] for up to 30 min, and included stationary cycling, walking, and jogging. The Borg scale was used throughout the session, aiming to maintain intensity at 12–15 (out of 20) [21].

The strength-based component included up to eight different resistance exercises targeting major muscle groups of the upper (i.e. chest press, bicep curls, tricep extensions, lat pull down, and shoulder press) and lower (i.e. squats, hamstring curl, calf raise, leg press, lunges) body. Body weight, dumbbell, and machine-based exercises were utilised and prescribed at 60–80% of one-repetition maximum (1RM) as determined during each patient’s baseline assessment. Prior to each session, patients’ treatment-related side effects and most recent blood test results were reviewed to determine their safety for exercise [22], with session modifications made if necessary.

### Outcome measures

Participants were assessed at baseline and at 10 weeks via a series of standardised tests. These tests were selected to provide insight into the cardiorespiratory fitness and functional capacity of this cohort undergoing cancer treatment. Normative data has been included as Supplementary Material (2) to allow for comparison of the cohort to age matched norms [23].

### Assessment of cardiorespiratory fitness

The primary outcome utilised cardiopulmonary exercise tests (CPEX) to evaluate submaximal peak oxygen consumption (VO_2_ peak). Participants’ resting heart rate and blood pressure were measured prior (Wahoo Tickr heart rate monitor, Wahoo Fitness LLC, GA, USA). Target heart rate (THR), calculated using the formula (220-age) [20] × 0.85, was used as criteria to terminate the test unless volitional exhaustion was met prior. The tests were conducted using a front access Exertech Ex-10 cycle ergometer (Repco Cycle Company, Huntingdale, Victoria, Australia) utilising a ramped protocol. Participants commenced cycling at 20 W for 1 min and increased by 20 W/min until they reached their THR or volitional exhaustion. At the end of each minute, heart rate and rate of perceived exertion (RPE) were recorded [21]. Participants completed a 3-min cool down following the test. During the test, participants were required to breathe through a mouthpiece connected to a computerised gas analysis system. The system included a ventilometer (Universal Ventilation Meter, Vacumed, Ventura, CA, USA) and oxygen and gas analysers (Ametek Applied Electrochemistry S-3A/1 and CD-3A, AEI Technologies, Pittsburgh, PA) which were used to calculate minute ventilation (V_E_), respiratory exchange ratios (RER), and oxygen and carbon dioxide in expired air at 15-s intervals. Values at the conclusion of the test were recorded in absolute (L/min) and relative (ml/kg/min) terms. Predicted peak oxygen consumption (V0_2_ peakpred) was extrapolated using the participants’ heart rate and V0_2_ from two submaximal stages from the test where a steady state heart rate between 115 and 150 beats per minute was recorded. The slope was calculated using the ratio of difference between the two submaximal V0_2_ measures and their corresponding heart rates and then used to extrapolate to predicted maximal heart rate [20, 24, 25]. The tests were conducted in a research setting by a trained sports scientist, blinded to group allocation [26].

### Assessment of physical function

Maximal lower and upper body strength was evaluated using standardised 1RM strength tests for leg press, chest press, and seated row exercises in accordance with ACSM guidelines [27]. Bilateral grip strength was assessed using a grip strength dynamometer (Jamar Plus Digital, Paterson Medical, IL, USA) with a rest time of 1 min between attempts. The best of three attempts was recorded. Functional upper and lower limb endurance was assessed using a 30-s push up and sit up test, as well as a five-repeated sit to stand (STS) test [27]. Participants with any known bone metastases were excluded from completing any tests that loaded the affected bone [10].

### Assessment of anthropometrics and body composition

Participant anthropometrics including height, weight, and body mass index (BMI) were assessed at each time point. Bone mineral density (BMD), total lean mass (LMM), total fat mass (FM), and body fat percentage (%FM) were assessed using dual energy X-ray absorptiometry (DEXA; Lunar Prodigy, GE Medical Systems, Madison, WI, USA).

### Assessment of patient-reported outcome measures

PROM were employed to capture changes in psychosocial variables, fatigue, and patient-reported physical activity. These included the European Organisation for Research and Treatment of Cancer Quality of Life questionnaire (EORTC QLQ-C30) [28], the Hospital Anxiety and Depression Score (HADS) [29], an age-specific version of the Paediatric Quality of Life Inventory (PedsQL), and multidimensional fatigue scale questionnaires [30–32], each of which have demonstrated good sensitivity to changes in clinical interventions or have specifically been validated in AYA [33, 34]. Self-reported physical activity was assessed using the International Physical Activity Questionnaire (IPAQ) [35]. Additionally, participants were required to complete an activity journal to capture any incidental exercise. However, despite ongoing efforts to ensure this was being undertaken, patient compliance in reporting detailed content was low which precluded this information from being used. All participants received standard medical care for their cancer treatment as well as their usual physical activity and diet throughout the intervention.

### Assessment of safety and feasibility

Eligibility, recruitment, adherence, and withdrawal rates were recorded throughout the trial and used to determine the feasibility of the intervention. Any adverse or serious adverse events were recorded and reported to appropriate HRECs.

### Sample size

A power analysis using PS Power and sample size calculations version 3.1.2 was performed to calculate the sample size required for this study, based on the primary outcome variable VO_2peak_ (ml kg min^−1^). In the absence of comparable published data in AYA patients, similar studies undertaken by Thorsteinsson et al. [36] and West et al. [37] in paediatric and adult patients demonstrated an effect size of 0.80. The standard deviation across multiple studies utilising VO_2peak_ as the primary outcome measure varies from 1.5 to 5.7 ml kg min^−1^ and, therefore, a median value (3.6 ml kg min^−1^) was utilised for this trials’ sample size calculation. Assuming a 5% significance level and a power of 0.8, a sample size of 36 patients (18 per group) will be required. An additional 10% has been included (*n* = 4) to allow for attrition or unusable data.

### Data and statistical analysis

Continuous data are presented as means and standard deviations, or medians and interquartile ranges (for skewed distributions). A linear mixed model regression was used to examine within and between group differences, adjusted for stratification variables. For within group findings, a negative value indicates a reduced score at 10 weeks, compared to baseline. When calculating between group differences, the corresponding baseline value was entered into the model along with the stratification variables. If the outcome data was skewed, transformations were attempted but were unsuccessful; therefore, nonparametric methods were utilised. For data with skewed distributions, the difference between time points were calculated for each subject to identify within group changes and the median of this difference with 95% CI is reported. The Wilcoxon signed-rank test was then used to analyse this within group change. For between group differences, change from baseline was calculated and analysed using the Mann–Whitney *U* test. Analyses for each outcome variable included only those participants with non-missing data at both time points. All data were analysed using Stata 16.1 (StataCorp, College Station, TX). Statistical significance was considered at *p* < 0.05.

## Results

Between December 2018 and January 2021, 43 participants were recruited and completed baseline assessments (Table 1), with no baseline group differences (*p*˃0.05).

**Table 1:** Characteristics of participants in the exercise (EG) and control (CG) groups, including prescribed treatments

Characteristic	EG (*n* = 21)	CG (*n* = 22)	*p* value
*Patient demographics*			
Age (years), mean (SD)	21.9 (3.0)	20.3 (2.7)	0.07
Males, *n* (%)	15 (68%)	12 (57%)	0.46
*Cancer diagnosis*			
Hodgkin lymphoma (*n*)	6	5	0.27
Sarcoma (*n*)	2	8	
CNS tumour (*n*)	6	5	
Germ cell tumour (*n*)	4	1	
Leukaemia (*n*)	2	2	
Melanoma (*n*)	1	0	
Burkitt lymphoma (*n*)	1	0	
*Treatment protocol*			
Low/moderate intensity			0.41
ABVD (*n*)	3	2	
Temozolomide (*n*)	4	5	
PCV (*n*)	1	1	
BEP (*n*)	4	1	
Ipilumamap/nivolumab (*n*)	1	0	
High intensity			
MAP (*n)*	0	2	
MAID (*n*)	1	0	
VDC/IE (*n*)	1	3	
ARST1431 (*n*)	0	3	
Escalated BEACOPP (*n*)	3	3	
CODOX M/IVAC (*n*)	1	0	
AML induction (7–3 Ida) + consolidation (5–2 ida) (*n*)	2	1	
ALL-09 (*n*)	0	1	
*Treatment intensity*			
Low/moderate intensity	13	9	0.29
High intensity	8	13	
*Radiation treatment*			
Radiation (*n*)	6	12	0.08
Dose of radiation (Gy), mean (SD)	17.05 (27.70)	31.26 (27.79)	0.10
Body surface area (m^2^), mean (SD)	1.88 (0.22)	1.91 (0.18)	0.49

### Cardiorespiratory fitness and physical function

Thirteen of the expected 86 (15%) CPEX were not completed as a result of medical contraindications, functional limitations, and/or restrictions due to the COVID-19 pandemic. All CRF and physical functioning data are presented in Table 2. No significant differences (*p* > 0.05) were evident between groups at 10 weeks for CPET or any physical functioning variables. With respect to Vo2_peak_, participants presented and remained within the 20–40th percentile and 10–20th percentile of normative values for 20–29 year old females and males, respectively. Additionally, participants in both groups presented with strength (1RM chest press and 1RM leg press) and functional (push ups and sit ups) outcomes within the 10–30th percentile when compared to age matched normative data (Supplementary file 2).While no significant improvement (*p*˃0.05) over the 10-week period was observed for any variable in the CG, the EG demonstrated a significant 21%, 10%, and 16% increase in 1RM leg press (*p* = 0.004), 1RM chest press (*p* = 0.032), and maximum push ups (*p* = 0.032), respectively.

**Table 2:** Comparison of cardiorespiratory fitness, strength, and physical functioning results including within and between group differences (mean ± SD)

	Exercise group (EG)				Control group (CG)				
Outcome	*n*	Baseline mean (SD)	10 weeks mean (SD)	Within group difference^a^ Contrast (95% CI) *p*value	*n*	Baseline mean (SD)	10 weeks mean (SD)	Within group difference^a^ Contrast (95% CI) *p*value	Between group difference^b^ Contrast (95% CI) *p*value
*Cardiorespiratory fitness*									
Vo2peakpred	17	34.0 (8.8)	35.9 (10.5)	1.9	16	28.7 (9.4)	27.8 (15.4)	− 0.9	1.2 (− 3.6, 6.1) p = 0.610
				(− 0.8, 4.5)				(− 4.5, 2.8)	
				*p* = 0.163				*p* = 0.637	
*Strength (1RM)*									
Leg press	20	102.8 (39.6)	124.1 (52.6)	21.4	14	97.9 (39.8)	103.0 (45.7)	5.2	15.8 (− 5.5, 37.1) *p* = 0.139
				(6.9, 35.8)				(− 5.6, 16.0)	
				***p* = 0.004**				*p* = 0.348	
Chest press	19	37.7 (14.3)	41.4 (15.8)	3.8	19	27.6 (15.3)	28.3 (15.5)	0.8	3.7 (− 1.1, 8.5) *p* = 0.127
				(0.3, 7.2)				(− 1.7, 3.2)	
				***p* = 0.032**				*p* = 0.545	
Seated row	20	55.5 (14.8)	59.0 (16.5)	3.5	19	46.6 (18.8)	46.8 (15.8)	0.3	4.7 (− 2.0, 11.4) *p* = 0.163
				(− 0.6, 7.6)				(− 4.9, 5.4)	
				*p* = 0.094				*p* = 0.920	
*Physical function*									
Hand grip (L)	20	37.6 (10.6)	38.0 (11.7)	0.4	19	29.2 (9.5)	28.6 (9.4)	− 0.6	1.4 (− 2.0, 4.8) *p* = 0.414
				(− 1.5, 2.3)				(− 2.8, 1.6)	
				*p* = 0.660				*p* = 0.599	
Hand grip (R)	20	40.3 (10.9)	40.5 (11.6)	0.2	19	31.3 (9.3)	31.4 (10.8)	0.1	0.5 (− 3.9, 4.9) *p* = 0.823
				(− 1.8, 2.2)				(− 3.1, 3.4)	
				*p* = 0.865				*p* = 0.935	
Push ups	18	16.9 (9.2)	19.6 (9.1)	2.7	12	11.7 (3.0)	13.0 (9.0)	1.3	2.0 (− 3.4, 7.5) *p* = 0.457
				(0.2, 5.2)				(− 3.3, 6.0)	
				***p* = 0.032**				*p* = 0.571	
Sit ups	18	14.5 (5.9)	14.7 (3.7)	0.2	16	12.4 (6.7)	12.8 (4.7)	0.4	1.3 (− 1.6, 4.3) *p* = 0.357
				(− 2.6, 2.9)				(− 3.4, 4.2)	
				*p* = 0.904				*p* = 0.846	
Sit to stand**	20	9.1	8.2	− 0.2 ^D^	19	12.5	10.7	− 0.5 ^D^	*p* = 0.961 ^R^
		(7.6, 10.2)	(7.1, 10.4)	(− 1.2, 0.2)		(9.0, 16.0)	(9.2, 13.5)	(− 2.2, 0.9)	
				*p* = 0.074 W				*p* = 0.233 W	

^**^Non parametric—between and within group differences for skewed distributions reporting the median and IQR

^a^Within group differences are adjusted for stratification

^b^Between group differences are adjusted for baseline and stratification

^D^Median difference with 95% CI

^W^Wilcoxon signed rank test (exact), it is not possible to adjust for stratification

^R^Rank sum (Mann–Whitney) non-parametric test using change from baseline scores, it is not possible to adjust for stratification

### Body composition

No change in either group for BMD or LMM was evident over time. However, both EG and CG demonstrated significant increases in FM% from baseline to 10 weeks (EG *p* = 0.029; CG *p* = 0.030). All body composition data are presented in Table 3.

**Table 3:** Comparison of body composition results including within and between group differences (mean ± SD)

	Exercise group (EG)				Control group (CG)				
Outcome	*n*	Baseline mean (SD)	10 weeks mean (SD)	Within group difference^a^ Contrast (95% CI) *p*value	*n*	Baseline mean (SD)	10 weeks mean (SD)	Within group difference^a^ Contrast (95% CI) *p*value	Between group difference^b^ Contrast (95% CI) *p*value
Lean muscle mass (total) (kg)	19	49.3 (7.9)	49.2 (8.5)	0.0	18	46.6 (10.2)	46.5 (11.1)	− 0.1	− 0.2 (− 1.8, 1.5) *p* = 0.830
				(− 0.9, 0.8)				(− 1.4, 1.2)	
				*p* = 0.931				*p* = 0.872	
Fat mass (total) (kg)	19	18.6 (13.2)	20.6 (12.3)	1.9	18	22.8 (8.4)	25.3 (10.5)	2.5	− 1.0 (− 4.6, 2.7) *p* = 0.593
				(0.0, 3.8)				(− 0.2, 5.1)	
				***p* = 0.046**				*p* = 0.069	
Fat mass (percent) (%)	19	25.5 (12.2)	27.9 (10.5)	2.4	18	32.6 (9.0)	35.3 (10.5)	2.7	− 1.3 (− 4.9, 2.3) *p* = 0.473
				(0.2, 4.6)				(0.3, 5.1)	
				***p* = 0.029**				***p* = 0.030**	
Bone mineral content (g)	19	2,693.1 (387.2)	2,679.3 (395.3)	− 13.8	18	2,630.0 (597.5)	2,611.0 (587.7)	− 18.9	1.9 (− 35.6, 39.3) *p* = 0.920
				(− 35.5, 7.9)				(− 46.3, 8.4)	
				*p* = 0.213				*p* = 0.175	
*Z*-score (BMD)	19	1.1 (0.8)	1.1 (0.7)	0.0	18	1.7 (1.3)	1.6 (1.3)	0.0	0.0 (− 0.3, 0.3) *p* = 0.981
				(− 0.1, 0.2)				(− 0.2, 0.2)	
				*p* = 0.673				*p* = 0.748	

^a^Within group differences are adjusted for stratification

^b^Between group differences are adjusted for baseline and stratification

### PROM

There were no significant group differences (*p* > 0.05) in patient-reported outcome measures (PROM) at 10 weeks (Table 4). However, the EG group reported a significant decrease in minutes of moderate-intensity physical activity completed per week (IPAQ MVPA *p* = 0.008) and an increase in minutes per day spent sedentary (IPAQ sedentary *p* = 0.046) from baseline to 10 weeks. Furthermore, the EG reported a significant decrease in their PedsQL Fatigue score (*p* = 0.014) indicating an increase in their fatigue from baseline. Additionally, results of the binary logistic regression indicated that minutes per day spent sedentary (IPAQ sedentary) significantly predicted PedsQL fatigue scores at 10 weeks (β =  − 0.028, t(36) =  − 2.517, *p* = 0.016). However, when minutes spent completing physical activity were categorised and measured against ACSM guidelines for PA in cancer survivors, the number of participants meeting these guidelines at 10 weeks among both EG and CG increased significantly from baseline (Table 5).

**Table 4:** Comparison of patient-reported outcome measures including within and between group differences (mean ± SD for parametric tests and median ± IQR for non-parametric tests)

Outcome (parametric)	*n*	Baseline mean (SD)	10 weeks mean (SD)	Within group difference ^a^ Contrast (95% CI) *p* value	*n*	Baseline mean (SD)	10 weeks mean (SD)	Within group difference ^a^ Contrast (95% CI) *p* value	Between group difference ^b^ Contrast (95% CI) *p* value
PEDS-QL									
Cancer	20	65.6 (16.0)	61.7 (12.6)	− 4.0	18	61.5 (18.3)	64.8 (14.4)	3.2	− 5.4 (− 12.2, 1.4) p = 0.118
				(− 10.7, 2.7)				(− 0.8, 7.3)	
				*p* = 0.246				*p* = 0.113	
Fatigue	20	58.2 (16.6)	50.7 (13.2)	− 7.5	18	51.7 (15.7)	53.0 (17.4)	1.4	− 5.8 (− 14.3, 2.7) *p* = 0.175
				(− 13.5, − 1.5)				(− 5.1, 7.8)	
				***p* = 0.014**				*p* = 0.678	
HADS									
Total	20	11.3 (7.9)	12.1 (6.3)	0.8	18	14.1 (7.1)	10.9 (6.3)	− 3.1	2.8 (− 0.3, 5.9) *p* = 0.072
				(− 1.6, 3.2)				(− 5.4, − 0.9)	
				*p* = 0.520				*p* = 0.006*	
Depression	20	5.3 (3.6)	5.0 (3.5)	− 0.3	18	5.8 (3.3)	5.1 (3.0)	− 0.8	0.2 (− 1.3, 1.7) *p* = 0.789
				(− 1.2, 0.6)				(− 2.1, 0.5)	
				*p* = 0.514				*p* = 0.248	
Anxiety	20	6.6 (4.6)	6.9 (3.8)	0.4	18	8.2 (4.3)	6.3 (3.3)	− 1.9	1.3 (− 0.8, 3.4) *p* = 0.224
				(− 1.6, 2.3)				(− 3.2, − 0.6)	
				*p* = 0.729				***p* = 0.005**	
IPAQ									
Sedentary	20	282.1 (138.3)	327.0 (146.3)	44.9	18	420.0 (170.9)	438.9 (252.7)	18.9	− 28.9 (− 165.5, 107.7) *p* = 0.670
				(0.8, 89.0)				(− 97.7, 135.5)	
				***p* = 0.046**				*p* = 0.751	
Outcome (non-parametric for skewed distributions)	*n*	Baseline median (IQR)	10 weeks median (IQR)	Within group median difference^D^ (95% CI) *p*value^W^	*n*	Baseline median (IQR**)**	10 weeks median (IQR)	Within group median difference^D^ (95% CI) *p*value^W^	Between group difference^R^ *p*value
IPAQ									
MVPA	20	870.0	240.0	− 360.0	18	105.0	160.0	− 15.0	*p* = 0.299
		(300.0, 1380.0)	(140.0, 465.0)	(− 853.7, − 14.0)		(0.0, 960.0)	(90.0, 340.0)	(− 483.6, 97.1)	
				***p* = 0.008**				*p* = 0.224	
EORTC-QLQC30									
Physical	19	93.0	87.0	0.0	16	87.0	87.0	1.5	*p* = 0.439
		(67.0, 100.0)	(80.0, 100.0)	(− 1.8, 6.3)		(63.0, 93.0)	(63.5, 98.0)	(− 2.9, 16.9)	
				*p* = 0.557				*p* = 0.277	
Role	19	67.0	67.0	0.0	17	67.0	67.0	0.0	*p* = 0.502
		(33.0, 83.0)	(50.0, 83.0)	(− 17.0, 33)		(33.0, 83.0)	(33.0, 67.0)	(− 16.0, 17)	
				*p* = 0.587				*p* = 0.958	
Emotional	19	67.0	68.0	0.0	17	75.0	75.0	0.0	
		(58.0, 100.0)	(50.0, 92.0)	(− 13.9, 3.1)		(50.0, 83.0)	(67.0, 92.0)	(0, 17.0)	*p* = 0.097
				*p* = 0.422				*p* = 0.074	
Cognitive	19	67.0	50.0	0.0	17	67.0	67.0	0.0	*p* = 0.782
		(50.0, 83.0)	(50.0, 67.0)	(− 21.9, 4.9)		(50.0, 83.0)	(50.0, 83.0)	(− 17.0, 17.0)	
				*p* = 0.482				*p* = 0.793	
Social	19	67.0	67.0	0.0	17	50.0	67.0	0.0	*p* = 0.660
		(50.0, 83.0)	(50.0, 100.0)	(− 17.0, 17.0)		(33.0, 67.0)	(50.0, 67.0)	(− 15.8, 17.0)	
				*p* = 0.954				*p* = 0.514	
Fatigue	19	56.0	56.0	11.0	17	44.0	56.0	11.0	*p* = 0.431
		(44.0, 67.0)	(44.0, 67.0)	(− 14.4, 11.3)		(22.0, 67.0)	(33.0, 67.0)	(0.0, 12.0)	
				*p* = 0.975				*p* = 0.141	
Nausea and vomiting	19	83.0	100.0	0.0	17	100.0	83.0	0.0	*p* = 0.243
		(67.0, 100.0)	(67.0, 100.0)	(− 4.9, 17.0)		(67.0, 100.0)	(67.0, 100.0)	(− 17.0, 0.0)	
				*p* = 0.180				*p* = 0.635	
Pain	19	83.0	83.0	0.0	17	67.0	83.0	0.0	*p* = 0.968
		(67.0, 100.0)	(67.0, 100.0)	(− 4.9, 17.0)		(33.0, 83.0)	(67.0, 100.0)	(0.0, 32.8)	
				*p* = 0.318				*p* = 0.332	
Global QOL	19	58.0	75.0	9.0	17	67.0	67.0	0.0	*p* = 0.219
		(50.0, 75.0)	(58.0, 92.0)	(0.0, 17.0)		(50.0, 75.0)	(50.0, 83.0)	(− 8.0, 15.9)	
				***p* = 0.011**				*p* = 0.552	

*PEDS-QL*, Paediatric Quality of Life Inventory with cancer specific module and fatigue module reported; *HADS*, Hospital Anxiety and Depression score with total score and anxiety and depression subscales reported; *IPAQ*, Global Physical Activity Questionnaire with Moderate-vigorous minutes physical activity (MVPA) and sedentary time reported; *EORTC-QLQC30*, European Organisation for the Research and Treatment of Cancer Quality of Life Instrument

^a^Within group differences are adjusted for stratification

^b^Between group differences are adjusted for baseline and stratification

^D^Median difference with 95% CI

^W^Wilcoxon signed rank test (exact), it is not possible to adjust for stratification

^R^Rank sum (Mann–Whitney) non-parametric test using change from baseline scores, it is not possible to adjust for stratification

**Table 5:** Comparison of patient reported physical activity data including within and between group differences

	Exercise group (*n*** = **21)	Control group (*n*** = *2***2)	*p* value ^a^
*Minutes of physical activity per week*			
Baseline			0.385
0–30 min	7	11	
31–90 min	2	0	
91–120 min	3	3	
121–150 min	1	0	
151 + minutes	8	8	
Total meeting PA guidelines ^**b**^	12	11	0.432
10-week assessment			0.385
0–30 min	1	4	
31–90 min	1	1	
91–120 min	0	2	
121–150 min	6	2	
151 + minutes	12	10	
Total meeting PA guidelines ^**b**^	18	14	0.087
Change from baseline to10 weeks for participants meeting PA guidelines (*p* value) ^c^	**0.009**	**0.016**	

^a^Pearson Chi-square: between group differences

^b^As per ACSM guidelines for cancer survivors.^10^

^c^Wilcoxon signed ranks test: within group differences over time

In terms of psychosocial well-being, despite no differences between groups at 10 weeks in the HADS total (*p* = 0.070), depression (*p* = 0.789), or anxiety (*p* = 0.224) scores, the CG reported a significant reduction in their HADS total (*p* = 0.006) and anxiety (*p* = 0.005) score over the 10-week period. Additionally, the EORTC-QLQC30 demonstrated no significant within or between group differences (*p* > 0.05) in the symptom-specific variables (physical, role, emotional, cognitive, social, fatigue, nausea, and vomiting and pain) over time. However, the EG reported a significant improvement in their Global QOL (*p* = 0.011) over the 10-week period although this was not associated with changes in any other outcomes over time (*p* > 0.05).

### Adherence, safety, and feasibility

Compliance to the study, protocol was evaluated relative to the number of exercise sessions attended and completion of assessments in both groups. Overall, 91% of participants completed the 10-week assessments (Fig. 1). Four participants (EG *n* = 1; CG *n* = 3) withdrew from the study prior to the 10-week assessment. EG participants completed 68% (m = 13.5, SD = 4.1) of the exercise sessions. Reasons for not competing exercise sessions included nausea and vomiting, safety restrictions, hospital admissions, restrictions due to COVID-19 pandemic, and logistical complications. The rigorous pre-exercise safety assessments resulted in the recording of several adverse events. Three participants required blood transfusions discovered by blood tests ordered as part of the study protocol. An additional three participants presented to exercise sessions with tachycardia (prior to exercising), requiring medical review. No adverse events were recorded during or following any exercise sessions as a direct result of the exercise prescription.

## Discussion

There is now strong evidence supporting the efficacy of exercise in mitigating treatment-related effects and improving survival outcomes in a range of cancer cohorts [38, 39]. However, research specifically within AYA cancer patients remains scarce [13], and given the distinctly, different physiological and psychosocial variables presenting in AYA caution should be taken in extrapolating outcomes from adult and paediatric populations. Despite the lack of statistical significance in study variables specifically between groups (EG and CG), the current study still demonstrated that a 10-week, supervised exercise intervention can reduce the functional decline in AYA undergoing cancer treatment. This work supports ACSM and Exercise and Sports Science Australia (ESSA) guidelines recommending exercise for cancer patients and demonstrates successful integration in AYA from diagnosis [9, 10].

CRF has been reported to decrease between 5 and 26% as a direct result of anti-cancer therapies and associated side effects [18, 36, 40]. Contrary to this, the current study demonstrated no change in CRF over the 10-week intervention period for both groups. This relative attenuation in CRF decline may have clinical implications on treatment tolerance with a recent systematic review and meta-analysis suggesting that CRF decline is associated with higher prevalence of acute and chronic treatment-related toxicities [18], albeit the results should be interpreted with caution in the current context given that this review was undertaken in adult-onset cancer patients and with a primary diagnosis of breast cancer (44% of the included review studies). The ability to maintain CRF is the ultimate goal for on-treatment patients, rather than a specific focus on improvement from baseline measures [9]. Therefore, through stabilisation of CRF in both groups, there may be an attenuation of treatment-related toxicities, requiring less rehabilitation post-treatment to regain normal functioning.

Previous research has reported significant decline in strength and physical function resulting from cancer and associated treatments [8, 36, 41]. The results from this study demonstrate that combined resistance and aerobic exercise can significantly improve upper (chest press and push up capacity) and lower body (leg press) strength, which was not apparent in the CG. Despite the EG only completing 68% (range 30–100%) of all exercise sessions, their mean 1RM leg press, chest press, and push ups all increased significantly (10–21%) over the 10-week period. Given the association between strength and ability to complete ADL it could be assumed that these results would contribute to the EG experiencing fewer limitations when completing ADL compared to CG throughout the intervention [42]. However, while the improvements in these variables may positively impact functional capacity in this cohort, it must be noted that both groups fitness, strength and functional data at baseline and following the intervention, remains below age-matched normative data and therefore needs to be addressed long term. Furthermore, it must be noted that only 52% of the EG group was compliant with the exercise protocol (> 80% attendance of exercise sessions); therefore, making it is difficult to interpret if these benefits would be greater with improved compliance.

An unexpected finding of the study was the lack of decline in physical functioning outcomes in the CG. As previously reported, cancer patients who participate in exercise studies are usually highly motivated to exercise, and as such, maintain or increase their exercise behaviour regardless of their group randomisation [43]. This is supported by the number of participants meeting physical activity guidelines for cancer survivors in both groups. At the 10-week assessment, 74% and 90% of the CG and EG, respectively, met the recommended > 90 min of moderate-intensity exercise per week [10]. This represented a 27% (CG) and 50% (EG) increase from baseline in those meeting the guidelines. Conversely, the average number of minutes of moderate and vigorous-intensity exercise (GPAQ MVPA) the EG completed weekly significantly decreased from baseline, demonstrating a large variability in this outcome measure across the EG cohort. This may also contribute to the lack of an observed difference between groups at 10 weeks. While this lack of decline in physical functioning in the CG was unexpected, from an ethical perspective, it is promising to see a relative attenuation of treatment-related decline that may potentially result from the simple enrolment, weekly monitoring of participants, and/or recording of incidental exercise in a weekly journal as part of the current study, let alone the motivation to exercise that may emerge from group discussion on physical activity.

Inconsistent with previous research, both the CG and EG groups in the current cohort demonstrated minimal body composition changes (LMM or BMD) over the 10-week period [44]. This attenuation of LMM loss may be attributed to the maintenance of some physical activity in both groups to 10 weeks. Additionally, the relatively short duration between assessments may not have been sufficient to capture significant changes in these variables, particularly BMD, which often shows the greatest changes 6–12 months post-diagnosis [45]. However, similar to previous research [46], the cohort demonstrated significant increases in %FM at 10 weeks regardless of group allocation. The fact that the EG and CG were spending approximately 5.5 and 7.5 sedentary hours per day, respectively, at the time of the 10-week assessment (significantly more for the EG group compared with their baseline reporting) may have contributed to their overall adiposity [47]. Furthermore, it must be noted that no dietary surveillance or reporting was sought as part of this study which may have contributed to the increased adiposity and warrants further investigation in the future.

Most importantly, a significant global improvement in QOL was observed in the EG over the 10-week period, which was not observed in the CG grouDespite this and, in contrast to previous findings [48], while there were no CG changes, the EG group demonstrated a significant increase in fatigue (PedsQL Fatigue) over time. This may be attributed to the significant increase in sedentary time (GPAQ sedentary) observed in the EG only, which has been reported to be a significant predictor of fatigue in this cohort (PedsQL Fatigue). However, this increase in fatigue in the EG was not supported by additional fatigue monitoring through the EORTC-QLQ-c30 subscale. Furthermore, this measure of fatigue does not distinguish between mental and physical fatigue (and/or the subsequent rest/recovery time that may be required as a result of exercise, rather than increased sedentary time resulting from general lethargy), highlighting a lack of sensitivity of these tools therefore making it difficult to draw conclusions on the true origin of fatigue or direction of this change. Given the 10-week timeframe, it may have been seen that exercising patients over a greater timeline build further fitness and increased tolerance to exercise, thereby reducing any sedentary time that may have been associated with post-exercise recovery. While no change was evident in global QOL in the CG, they demonstrated significant improvements in psychosocial well-being (HADS total and HADS anxiety) at 10 weeks. No change was evident in the EG and both groups were not statistically different at 10 weeks. This potentially reflects the heightened distress of the CG at baseline, which normalised relative to the EC with the ongoing support offered through enrolment in this study. Additionally, it may be reflective of the CG experiencing a reduction in symptomatology such as pain relative to the commencement of their treatment thus impacting their psychosocial well-being. We are aware of the danger of over-interpreting results in the small numbers of patients on study; however, RCTs of large numbers of AYA with specific cancers and specific treatment regimens are unlikely to be possible.

### Limitations

While this study is the first to report on the impact of exercise specifically among AYA during treatment, several limitations are acknowledged. The study design resulted in the inability to blind study participants to group allocations, therefore potentially contaminating the CG. Furthermore, a selection bias in terms of the nature of participants to be motivated exercisers when consenting to join an exercise study may confound the results. Subsequent to this, participants in the CG were contacted weekly to monitor treatment-related toxicities and ensure compliance to the study protocol, thus potentially impacting their exercise behaviour. Additionally, four participants withdrew from the study prior to the 10-week assessment due to non-compliance to the study protocol and/or disease progression. Furthermore, the study was powered based on the primary outcome of Vo2peak, of which a number of participants were unable to complete due to safety restrictions and as such this attrition and non-compliance reduced the strength of the sample and may have impacted study results. Finally, the COVID-19 pandemic impacted recruitment, exercise sessions (and possibly adherence), data collection (including assessment timeframes), and compliance to study protocol.

## Conclusions

This study demonstrated that the 10-week supervised exercise program (EG) resulted in superior strength and QOL outcomes in AYA undergoing treatment for cancer, compared with usual care (CG). Unexpectedly, there was no decline in the CG group suggesting potential benefits gained from the enrolment in the exercise-based study (in the form of maintenance of independent physical activity and function), with weekly monitoring. Furthermore, investigation into the specific benefits of an independent exercise program design with frequent monitoring is warranted.

## Supplementary Information

Below is the link to the electronic supplementary material.

Supplementary file1 (DOCX 13 KB)

Supplementary file2 (DOCX 18 KB)

## Data Availability

Available on request.

## Declarations

### Disclaimer

Funders have had and will have no role in the study design, data collection, analysis and interpretation of data, or submission for publication processes.
