# Supplementary material for: A randomised controlled trial investigating the ability for supervised exercise to reduce treatment-related decline in adolescent and young adult cancer patients
Source: Support Care Cancer. 2022 Jul 6;30(10):8159–71. doi: 10.1007/s00520-022-07217-w (PMC9257117; doi:10.1007/s00520-022-07217-w)
Supplement: Supplementary file 1 — Supplementary file1 (DOCX 13 KB) [file 520_2022_7217_MOESM1_ESM.docx]

Supplementary table 1. Stratification strategy of most common adolescent and young adult cancer diagnoses based on treatment regimen.

| **Low/Moderate intensity treatment diagnoses** | **High intensity treatment diagnoses** |
| --- | --- |
| Hodgkin lymphoma  Germ cell tumours  Gynaecological tumours  Non-Hodgkin lymphoma (excluding Burkitt)  Melanoma | Acute lymphoblastic leukaemia  Lymphoblastic lymphoma  Burkitt lymphoma  Soft tissue and bone sarcoma  Squamous cell carcinoma of the head and neck |
