# Supplementary material for: A randomised controlled trial investigating the ability for supervised exercise to reduce treatment-related decline in adolescent and young adult cancer patients
Source: Support Care Cancer. 2022 Jul 6;30(10):8159–71. doi: 10.1007/s00520-022-07217-w (PMC9257117; doi:10.1007/s00520-022-07217-w)
Supplement: Supplementary file 2 — Supplementary file2 (DOCX 18 KB) [file 520_2022_7217_MOESM2_ESM.docx]

**Supplementary Material 2 – Physical fitness normative data.**

Normative data presented for strength and functional tests for 20-29 year old male and females. All data presented as a range from the 10^th^ to 90^th^ percentile.

|  | **Males** | **Female** |
| --- | --- | --- |
| Vo2peak (ml.kg.min^-1^) ^a^ | 32.1-61.8 | 23.9-51.3 |
| Maximal push ups ^a^ | 14-41 | 5-30 |
| Maximal sit ups ^a^ | 16-75 | 13-50 |
| 1RM chest press* ^b^ | 0.80-1.48 | 0.48-0.90 |
| 1RM leg press* ^b^ | 1.51-2.27 | 1.14-1.82 |

*Calculated as a ratio of weight lifted (kg) ÷ body weight (kg) and presented from 10-90^th^ percentile.

**Example:**

*Leg press (At baseline)*

Mean male weight (kg): 74.03

Mean male leg press (kg): 104.63kg

Ratio: 1.41

Conclusion: Participant below 10^th^ percentile.

*Chest press (At baseline)*

Mean female weight (kg): 74.77kg

Mean female bench press (kg): 18.90

Ratio: 0.25

Conclusion: Participant below 10^th^ percentile.

References

^a^ Barton, A., Zabriskie, H. A., Moxley, J., & Blackshear, T. (2021). Normative Fitness in Undergraduate Exercise Science Students: How Do They Compare?. *Quest*, 1-15.

^b^ Liguori, G., & American College of Sports Medicine. (2020). *ACSM's guidelines for exercise testing and prescription*. Lippincott Williams & Wilkins.
